# Assessment of viral methylation levels for high risk HPV types by newly designed consensus primers PCR and pyrosequencing

**DOI:** 10.1371/journal.pone.0194619

**Published:** 2018-03-26

**Authors:** Anna Gillio-Tos, Valentina Fiano, Chiara Grasso, Morena Trevisan, Silvia Gori, Alessandra Mongia, Laura De Marco, Guglielmo Ronco, and the New Technologies for Cervical Cancer Screening (NTCC) Working Group

**Affiliations:** 1 Cancer Epidemiology Unit–C.E.R.M.S, Department of Medical Sciences, University of Turin, Turin, Italy; 2 Immunology and Diagnostic Molecular Oncology Unit, Veneto Institute of Oncology IOV IRCCS, Padua, Italy; 3 Regional Cancer Prevention Laboratory—Oncological Network, Prevention and Research Institute (ISPRO), Florence, Italy; 4 Cancer Epidemiology–CERMS, City of Health and Science Hospital and CPO, Turin, Italy; 5 Centre for Oncologic Prevention, Turin, Italy; Fondazione IRCCS Istituto Nazionale dei Tumori, ITALY

## Abstract

**Background:**

Measuring viral DNA methylation in human papillomavirus (HPV) infected women showed promise for accurate detection of high-grade cervical lesions and cancer. Methylation status has been widely investigated for HPV16, sporadically for other HPV types.

**Methods:**

Objective of this methodological study was to set up molecular methods to test the methylation levels in the twelve oncogenic HPV types by pyrosequencing, minimizing the number of HPV type-specific PCR protocols. Target CpGs were selected on the HPV L1 (two regions, L1 I and L1 II) and L2 genes.

Study samples included DNA stored at Turin, Italy, purified by cervical cells collected in Standard Transport Medium or PreservCyt from women who participated in two studies (N = 126 and 140) nested within the regional organized screening programme.

PCR consensus primers were designed by PyroMark Assay Design software to be suitable for amplification of many different oncogenic HPV types.

**Results:**

Generation of consensus primers was successful for L1 I and II regions, unsuccessful for L2 region, for which HPV type-specific primers remained necessary. The difference between replicated tests on the same sample was ≤4% in 88%, 77% and 91% of cases when targeting the L1 I, L1 II and L2 regions, respectively. The corresponding intra-class correlation coefficients (ICC) were 0.94, 0.87 and 0.97 respectively. When comparing methylation measures based on consensus and type-specific primers, ICC was 0.97 for the L1 I region and 0.99 the for L1 II region.

**Conclusions:**

The proposed protocols, applying consensus primers suitable to amplify the oncogenic HPV types and minimize the number of PCR reactions, represent a promising tool to quantify viral methylation in women positive for any high risk HPV type.

**Impact:**

Potential application of these methylation protocols in screening settings can be explored to identify women with high probability of progression to high grade lesions.

## Introduction

Persistent infection by oncogenic high risk human papillomavirus (hrHPV) types drives the development of high-grade cervical intraepithelial neoplasia (hgCIN) which may, if untreated, progress to invasive cervical cancer.

In hrHPV positive women, measuring DNA methylation in the HPV genome has shown promise for accurate detection of hgCIN and cancer [1–9]. The HPV genome is poorly methylated at the time of cell infection and it does not harbour methylating enzymes but can be affected by methylation events driven by the host cell DNA methyltransferases [10,11]. At present, methylation status has been investigated mostly in HPV16 and to a lesser extent in the remaining oncogenic HPV types [2,3,12–15]. Most studies employed whole genome sequencing, with the aim of assessing the methylation status of the majority of viral CpGs, which are widespread along the HPV genome and poorly clustered in islands [16]. This approach provided evidence that methylation events, which represent a defence mechanism by the host cell to silence viral DNA [17], largely involve most of the viral CpGs, although single CpG sites can be methylated at different levels. Mirabello and collaborators [4] showed indeed that most of the CpG sites in the HPV16 genome appear to be methylated in a coordinated fashion. Previous results suggested that selected CpGs, located in the HPV late regions L1 and L2, are associated to current or future high grade CIN detection [18,19]. In particular, this association resulted very strong for some CpGs located in the L1 and L2 capsid genes consistently across studies [2,4,5,13–15,20,21]. To our knowledge, however, among these studies, only a recent one [15] focused on the evaluation in cervical scrape samples of HPV methylation levels in all the twelve oncogenic HPV types (16, 18, 31, 33, 35, 39, 45, 51, 52, 56, 58, 59) [22], by using a next generation sequencing approach.

A commonly used method for assessing methylation status of DNA is pyrosequencing. This entails a preliminary amplification of bisulfite-modified DNA, until now performed by type-specific primers. The present paper reports about the development of consensus primers for multiple HPV genotypes and their use to evaluate methylation status of the L1 and L2 viral regions by pyrosequencing. This approach is able to minimize the number of PCR protocols needed to investigate the methylation status in the twelve hrHPV types.

We report here about its development and analytical validation, including the reproducibility of methylation values on the same sample and the consistency of such values with those obtained by type-specific primers. The association of such values with the current or future detection of high-grade cervical intraepithelial neoplasia (CIN) is not included. This would take as itself the space of another full article.

## Methods

### General study design

CpG sites were selected for HPV16, based on their location in the L1 or L2 regions and the strong reported association of their methylation with hgCIN occurrence. For the remaining hrHPV types the CpGs closest to those selected for HPV16 were identified. Type-specific and consensus primers for the defined sequences were generated and checked to avoid artefact sequences. PCR and pyrosequencing conditions were optimised. Consensus primers were validated, under such optimised conditions, as for the reproducibility of the estimated methylation and its consistency with that obtained by applying type-specific primers.

### Study samples

We used two series of DNA samples, obtained from women participating to: (1) the NTCC study (a randomised controlled trial which provided evidence of greater efficacy of HPV-based than cytology-based screening in preventing invasive cervical cancer [23,24]) and (2) the HPV pilot project, subsequently conducted to evaluate the routine application of HPV based screening [25]. Both studies were nested within the organized screening programme of Turin, Italy. Both had been approved by the local Ethical Committee of Turin (Comitato Etico Interaziendale A.O.U. Città della Salute e della Scienza di Torino—A.O. Ordine Mauriziano—A.S.L. TO1) along with the storage of residual cervical cell samples for future molecular investigations after written informed consent. DNA had been extracted from cervical cell samples taken at enrolment through the QIAamp DNA Mini Kit (Qiagen, Hilden, Germany) according to manufacturer instructions and genotyped for hrHPV types by the Digene HPV genotyping RH kit (Qiagen) as previously described [26].

Only samples from HPV positive women with infection by a single hrHPV type at the screening test were included.

DNA samples from the HPV pilot project (N = 126) were used to develop and set up the novel methylation assays. Ten to thirteen samples for each hrHPV type were randomly retrieved from storage. They had been extracted from cervical scrapes collected in STM (Standard Transport Medium, Qiagen) and had been positive to the Hybrid Capture 2 (HC2, Qiagen) HPV screening test.

DNA samples from the NTCC trial (Turin centre) (N = 140) were used to assess the reproducibility of the newly developed quantitative methylation assays. They had been extracted from cervical scrapes collected either in STM or in PreservCyt (Hologic/Gen-Probe, San Diego, CA) and had been HC2 positive. As they had been already used to analyse methylation status in selected host cell genes (data under evaluation) residual bisulfite modified DNA stored at -80°C was used for the current reproducibility evaluation, with each sample tested twice. For each hrHPV type five to twenty-one samples were randomly retrieved from bisulfite modified DNA storage.

### CpG selection

As methylation events had been suggested to involve most of the HPV CpGs in hgCIN [2–7,11], we skipped the whole viral genome sequencing and focused on specific CpG sites reported with statistically significant odds ratios (ORs).

For all the HPV oncogenic types the CpG sites to analyse were selected on the L1 and L2 capsid genes on the basis of the following considerations: i) in some HPV types (e.g. HPV18) methylation events poorly involve other viral genes, such as the URR (untranslated regulatory region) and E6 [27]; ii) the results of the studies on the association between methylation of URR and hgCIN were controversial [9,28]; iii) HPV late genes show higher sequence homology among types, which could make the generation of consensus primers easier. Furthermore, selection was focused on CpG sites that had been reported to be hypermethylated in association with hgCIN (with OR ≥2 or p <0.05) in at least two published studies.

Reference sequences and CpG positions for each HPV type were found at www.ncbi.nlm.nih.gov web site and are listed in Table 1.

**Table 1:** Reference sequences for the twelve oncogenic HPV types and CpG positions.

HPV type	Reference sequence	CpG position						
		L1 I^a^				L1 II^b^		L2
**16**	NCBI: NC_001526.2	5601	5606	5609	5616	6457		4261
**18**	GenBank: KC470224.1 (L1) EF202151.1 (L2)	5599		5616		6433		4268
**31**	GenBank: J04353.1	5518	5521	5524	5530	6498		4195
**33**	GenBank: M12732.1	5557	5560	5566	5572	6409		4234
**35**	GenBank: M74117.1	5540	5543	5546	5553	6519		4208
**39**	GenBank: KC470245.1	5682				6513		4301
**45**	GenBank: KC470260.1	5620		5636		6463		4260
**51**	GenBank: GQ487711.1 (L1) KF436879.1 (L2)	13				848		4158
**52**	GenBank: HQ537750.1	5613	5616	5622	5628	6450		4290
**56**	GenBank: EF177179.1	5561	5567	5570	5576	6475	6496	4247
**58**	GenBank: GI222386	5606	5609	5615	5621	6447	6458	4268
**59**	GenBank: KC470266.1	5618				6452		4255

^a^ Some HPV types do not have all the four CpGs found in the HPV16 reference sequence, but only two (HPV18, 45) or one (HPV39, 51, 59)

^b^ Some HPV types (HPV56, 58) have two CpGs close to the CpGs selected in HPV16 sequence. For these types the average between the methylation values of two CpGs can be considered.

HPV16 genome sequence was used as reference and three regions, hereafter denoted as L1 I (CpG 5601, 5606, 5609, 5616) [4,5,13–15,20,21], L1 II (CpG 6457) [4,14] and L2 (CpG 4261) [2,4,5,15,20], were selected.

For non-HPV16 oncogenic types, CpG sites were identified through the multiple sequence alignment tool Clustal Omega (www.ebi.ac.uk/ Tools/msa/clustalo/), which allowed visualizing the sequence homology and identifying, along each HPV type sequence, the CpG closest to each selected HPV16 CpG. Sequence alignment was performed following conversion to bisulfite modified sequences by the PyroMark Assay Design 2.0. software (Qiagen). The correct format to fit Clustal Omega tool was obtained by the Emboss Seqret software (www.ebi.ac.uk/Tools/sfc/emboss seqret).

For each hrHPV type, the CpGs aligned to the selected HPV16 CpGs were chosen for analysis. In case of imperfect alignment, nearby CpGs were selected. When all twelve hrHPV sequences were aligned homology was poor. Substantial improvement in sequence homology was obtained by grouping types in two families, mostly based on their phylogenetic affinity and hereafter defined as “16 family” (HPV 16, 31, 33, 35, 52, 56, 58) and “18 family” (HPV 18, 39, 45, 51, 59).

### Generation of primers

Primers for the selected target regions L1 I, L1 II and L2 of the “16 family” and “18 family” were designed through the PyroMark Assay Design 2.0 software (Qiagen). Software stringent criteria, such as the mandatory requirement to avoid primer overlap of CpG sites and, when possible, to avoid homopolymers and the generation of amplicons longer than 300 bp, were taken into account. Sets of three primers, two for the amplification of the sequence that contains the selected CpGs and one for its sequencing, were generated for each hrHPV type.

Quality checks were performed throughout pyrosequencing to avoid artefact sequences: either the amplicon alone, or the biotinylated primer or the sequencing primer were combined with the annealing buffer and tested, expecting no sequencing. For the same purpose, each biotinylated primer was combined with the sequencing primer. Moreover, primers were tested on hrHPV negative samples to assess their specificity for the HPV genome and exclude cross-reaction with host cell genome. Primers were first tested on samples containing the specific hrHPV type. Further, within each HPV family, each forward primer was coupled with each reverse primer and tested for amplification of all the types included in the family, in the aim to identify potential consensus primers favoured by sequence homology. Similarly, each sequencing primer was tested by pyrosequencing with amplicons from all the hrHPV types of the same family. Optimization of PCR and pyrosequencing was performed both on DNA samples and DNA controls (SiHa and HeLa cell lines; HPV16 and HPV18 synthetic plasmids, Medical System, Genoa, Italy).

### Bisulfite modification

The methylation status of a DNA sequence can be determined after DNA treatment with sodium bisulfite, which deaminates unmethylated cytosine residues on single-stranded DNA molecules and converts them to uracils, whereas 5-methyl cytosines remain protected from conversion. DNA polymerase in PCR driven assays converts the uracil residues of bisulfite modified DNA to thymidines. Different DNA strands, distinguishable by sequencing, are therefore generated from methylated and unmethylated CpGs.

DNA from clinical samples (≤ 1 μg), as well as from SiHa and HeLa cell line, used as methylation positive controls for the HPV16 and HPV18 family respectively, underwent bisulfite modification using the Epitect Bisulfite Kit (Qiagen) according to the manufacturer’s instructions, except for the incubation time extended to 16 h, optimized in line with suggestions by Izzi and coll. [29].

Synthetic HPV16 and HPV18 plasmidic DNA containing the complete genomes (Medical System, 1 μg) were used as unmethylated controls. These synthetic HPV plasmids were also used as fully methylated controls after treatment with CpG methylase (M.SssI, Zymo Research, Irvine, CA, USA) according to the manufacturer’s instructions. Both CpG methylase-treated and -untreated HPV plasmids underwent two cycles of bisulfite modification with incubation time extended to 16 h to achieve a complete conversion. Modified DNA was used immediately or stored at -80°C until methylation assessment.

### Methylation analysis

Methylation assays were performed by pyrosequencing onto a PyroMark Q24 MDx system (Qiagen). For regions L1 I and L1 II preliminary PCR reactions were performed in a total volume of 35 μl containing 1X PCR buffer, 2mM MgCl_2_, 0.8 mM dNTPs, 0.5 μM of each primer, 1.75U Taq polymerase and 2 μl of bisulfte modified DNA. The cycling profile was as follows: 95°C for 10 min followed by 45 cycles of denaturation at 95°C for 30 s, annealing for 1 min at the specific temperature set for each hrHPV type (Table 2), extension at 72°C for 1 min. Extension at 72°C for 10 min was finally performed.

**Table 2:** L1 I and L1 II. Primers sequences and annealing temperatures.

Target Region	HPV type	PCR primers and assay conditions			Pyrosequencing primers	
		PCR primer ^a^	Ampl. bp	PCR ann. T	Sequencing^a^ primer	HPV strand
**L1 I ^b^**	**16,31,33,35,52,58**	Forward GATATTTGTAAAAAAATATGGAA Reverse bio-AATAACTTTTATTTACATCCTAATTAT	78	45	All types GATATTTGTAAAAAAATATGGAA	Lower
	**56**	Forward TGAAATAGGTGTTGGAGGTAGAT Reverse bio-CCCATAATATATATATACAAAAATCCTCCT	143	62	Type 56 GTTATTTGTAAAAAAATAGGGAA	Lower
	**18**	Forward TTTATATTGGTATGTAGAAATTTTAGG Reverse bio-CCCTATTTTTTTACAAATAACTTTATAAC	229	50	Type 18 TTATAGAAGGTGGTGGAAGAT	Lower
	**39,45,51,59**	Forward TTTATATTGATATGAGGATATTTTAGG Reverse bio- CCCTATTTTTTTACAAATAACTTTATAAC	229	50	Type 39 AAATATATTATGTTGTTATTAGAT Type 45 TTATAGAAGGTGGTGGAAGAT Type 51 ATATATTTTGTTGTTATTAGTG Type 59 GTAGATATATTTTGTTGTTATTAGA	Lower
**L1 II ^c^**	**16,33,35,52,56,58**	Forward GTTAGATATTTTTTTAATAGGG Reverse bio-ATTACCCCAACAAATACCATTA	207–219	57	Type 16 TGTTGGTGAAAATGTATTAG Type 33 GGTATATTAGGAGAGGTTGT Type 35 ATATTTAATAAATTATATTGGTTGT Type 52 GTTAGATATTTTTTTAATAGGG Type 56 GAGAATTTTTTTTTAGTTTTGTATA Type 58 TAGGGTTGGAAAATTTGG	Upper
	**31**	Forward GATGTATAAATATTTAATAAATTA Reverse bio- ATTACCCCAACAAATACCATTA	75	45	Type 31 GATGTATAAATATTTAATAAATTA	Upper
	**18,51**	Forward GTTTGTAGATTTTTATGGGGATTTTATG Reverse bio- ACCAACAAATACCATTATTATAACCCT	266	50	Type 18 ATATATTAAAGGTATAGGTATG	Upper
	**39,45,59**	Forward TTGTAAGATATTTTTGGAAT Reverse bio- ACCAACAAATACCATTATTATAACCCT	210	55	Type 39 AATTGTATATTAAGGGTA Type 45 AGATTTATATATTAAAGGTATTAG Type 51 ATTATTATATTAAGGGTAGTGGTA Type 59 GTTATATATTAAAGGTATTGA	Upper

^a^ Bisulfite modified sequences

^b^ L1 I. HPV L1 gene. Reference HPV16 CpG: 5601, 5606, 5609, 5616

^c^ L1 II. HPV L1 gene. Reference HPV16 CpG: 6457

For the L2 region PCRs were performed employing the PyroMark PCR Kit (Qiagen) following manufacturer’s instructions, except for the annealing temperature set for each hrHPV type as listed in Table 3.

**Table 3:** L2. Primers sequences and annealing temperatures.

Target Region	HPV type	PCR primers and assay conditions			Pyrosequencing primers	
		PCR primer ^a^	Ampl. bp	PCR ann. T	Sequencing ^a^ primer	HPV strand
**L2** ^b^	16	Forward GTTAGGTGGATATGTATTTGTT Reverse bio- ATACCATTATTTTTAATACATACACATAC	276	58	ATGTTTTATAAAGTTGGG	Lower
	18	Forward AGGTTGGTTTATATAGTGTATTGT Reverse bio- ATACCTATACCAAATCCACC	263	56	TTGTATTTTTATAATAAAATTATGG	Upper
	31	Forward TGGATAAGTATTTGTTGTTTTATATGTT Reverse bio- CCATTATTTATAATTCATACACATACAT	235	50	TTTGATATAATTGTGTAGTAG	Lower
	33	Forward GGTTTTGTATGTTTGGTATAGTT Reverse bio- ACTAATAAATACCTTTATATTTTAACAAT	106	50	GTATAGTTGTGTTGTAGATG	Lower
	35	Forward TGGATAAGTTTTTGTAGTTTTGTAA Reverse bio- AATAAATTAATCACATAATATAACCATAC	112	48	TAGTTGTGTTGTAGATG	Lower
	39	Forward bio- ATAGGTTTTATATAGGTTAGTTGTAGA Reverse ACCACACCATTAAAAATATTTTATA	226	56	AATAAACATAATTTCCCACC	Lower
	45	Forward bio- TATGTTTTATATAAGTTAGTTGTAGA Reverse ACATCCCCATTAACAACATTTACTA	269	56	AACCATAATATCCCACC	Lower
	51	Forward GTTTTTGGTTTGTTGTTGTAATATTTTAA Reverse bio- AACCCACTCCACTATAATATTTTATC	87	56	TTGTGATTAAATATGGTGG	Upper
	52	Forward GTTAGAGGTTTTGTATGTTTGATATAG Reverse bio- ATTTTCCAATATTTTATATTCACTATCAT	239	50	ATATAGTTGTGTTGTAGAA	Lower
	56	Forward TTGGATATGTATTAGATAATTTATATGTT Reverse bio- ACCACATCCTTTTTTAATACATTTATA	209	48	ATATAGTTGTGTTGTAGA	Lower
	58	Forward GGTTTTGTATGTTTGGTAAAGTT Reverse bio- CATACACAATACTTAACCCAAC	221	58	GGTAAAGTTGTGTAGTAGA	Lower
	59	Forward GGGTATGTATTTGTTTGTTTGTAG Reverse bio-AATACTATATACCCATACAATACTATCC	221	56	AAGTTTGTTGTTGAGG	Lower

^**a**^ Bisulfite modified sequences

^b^ L2. HPV L2 gene. Reference HPV16 CpG: 4261

Two μl of bisulfite modified DNA were added to the amplification mix.

Amplicons were analyzed by gel electrophoresis on a 2% agarose gel stained with DNA intercalating dye (e.g. Syber Safe) and visualized by ultraviolet trans-illumination. Twenty μl of PCR product were added, in a 24-well microplate, to 60 μl of a mix composed by 18 μl distilled water, 40 μl binding buffer pH 7.6 (containing 10mM Tris-HCl, 2 M NaCl, 1mM EDTA, 0.1% Tween 20), and 2 μl of sepharose beads covered by streptavidin, and incubated under shaking. PCR products were then washed with ethanol 70%, denatured with NaOH 0.2 M and re-washed with Tris-Acetate 10 mM pH 7.

Pyrosequencing was performed in a total volume of 25 μl, including 24.85 μl of annealing buffer (20 mM Tris-Acetate, 5 mM MgAc_2_) and 0.15 μl of 50 μM type-specific sequencing primer (final concentration 0.3 μM). Assays were created according to the manufacturer’s instructions.

The nucleotide dispensation order was outlined by software Q24 2.0 for each HPV genotype. Pyrosequencing reactions were performed in CpG mode (software Q24 version 2.0) which shows high performance in quantitative evaluation of the proportion of methylated cytosines at each CpG site through the C/(C+T) ratio [30]. Based on the correspondence of the sequence to that expected for each genotype and of the quantity of material available, the quality of the result at each position is classified by the PyroMark CpG mode software as “passed” (valid/acceptable), “check” (interpretation by operators needed) or “failed” (pyrogram not interpretable). The height of the peak was proportional to the number of sequenced amplicons.

Examples of pyrograms are available in the supplementary data, S1 Fig.

### Validation

#### Consistency with type specific PCR

Comparison was carried out for L1 I and L1 II regions by testing with the two different assays a total of 90 samples from the pilot project (S1 Table), some of them used for both regions. Comparison was limited to 8 frequent HPV types (HPV 16, 18, 31, 33, 45, 52, 56 and 58), the seven hrHPV types of the nonavalent vaccine causing >70% of hgCIN worldwide [31], and the HPV56. The methylation on the L1 I and L1 II regions was compared separately, considering the mean percentage of CpGs measured as methylated in each region.

#### Reproducibility of methylation estimates

We evaluated the protocols performance by applying the assays to a series of samples (N = 140) from the NTCC Turin cohort that included long-stored DNA. Half of them had been obtained from cells originally collected not in STM but in a different transport medium (PreservCyt). Bisulfite modified DNA samples were tested. To assess the reproducibility of methylation, the procedure was replicated by performing for each sample a second PCR run along with the subsequent correspondent pyrosequencing assay. Again, methylation on the L1 I, L1 II and L2 regions was compared separately, considering the mean percentage of CpGs measured as methylated in each region and afterwards computing the difference between the mean percent methylation. Because we used residual modified samples from previous analysis on human genes, some samples did not have sufficient material to perform replicates on all the three target regions. Therefore, replicates on the L2 region were 105 instead of 140.

### Statistical methods

The agreement between the percentage of methylated CpGs measured by testing two aliquots of the same sample with consensus and type specific primers and that obtained by replicate testing with consensus primers were evaluated by the intra-class correlation coefficient (ICC). For this purpose, we used a log transformation of methylation values, so to normalise their distribution (Shapiro-Wilk test p = 0.55 for type specific and p = 0.21 for consensus primers in the L1 I region; p = 0.65 and p = 0.69 respectively for the L1 II region). ICC does not assume different accuracy of compared variables and takes into account systematic differences between measurements (i.e. considers the alignment on a line with slope = 1). Wilcoxon test was used to compare methylation levels by cytology groups.

## Results

### Identification of consensus primers

Sequence variability was found not only among types but also within the same type, and several primers had to be designed and tested before setting up the final assays. At first primers dedicated to each hrHPV type were generated. The sequences of HPV type-specific primers for the L1 I and L1 II regions, with the corresponding PCR profile, are listed in S2 Table. Afterwards, consensus primers for analysing methylation in the L1 I and L1 II HPV regions were obtained for groups of hrHPV types within both HPV families (Table 2). For the consensus primers of L1 I region PCR conditions were identified, working efficiently at annealing temperature of 45°C for PCR targeting HPV16, 31, 33, 35, 52, 58 of the “16 family”, and of 50°C for HPV45, 39, 51, 59 of the “18 family”. With these PCR profiles consensus primers were however not efficient for HPV56 and HPV18: for those types, dedicated primers and annealing temperature were maintained.

Similarly, for the L1 II region consensus primers and annealing temperature were set for PCR targeting HPV16, 33, 35, 52, 56, 58 of the “16 family” and HPV18, 51 and 45, 39, 59 of the “18 family”. For HPV31 consensus primers were not efficient, and dedicated primers and annealing temperature were maintained.

Generation of consensus primers for the L2 region was not successful, and dedicated primers for each HPV type were maintained, along with specific annealing temperatures (Table 3).

Methylation levels for each region and the corresponding PyroMark Q24 score for the 126 tested DNA samples (Pilot project) are reported at supplementary S3 Table. No “failed” score was assigned by the PyroMark software. “Check” scores were due to a low amount of viral DNA in the sample, resulting in low peak height. Therefore, they did not spoil the reliability of the quantitative result or the efficiency of the assay.

### Consistency with type specific PCR

**Fig 1:**
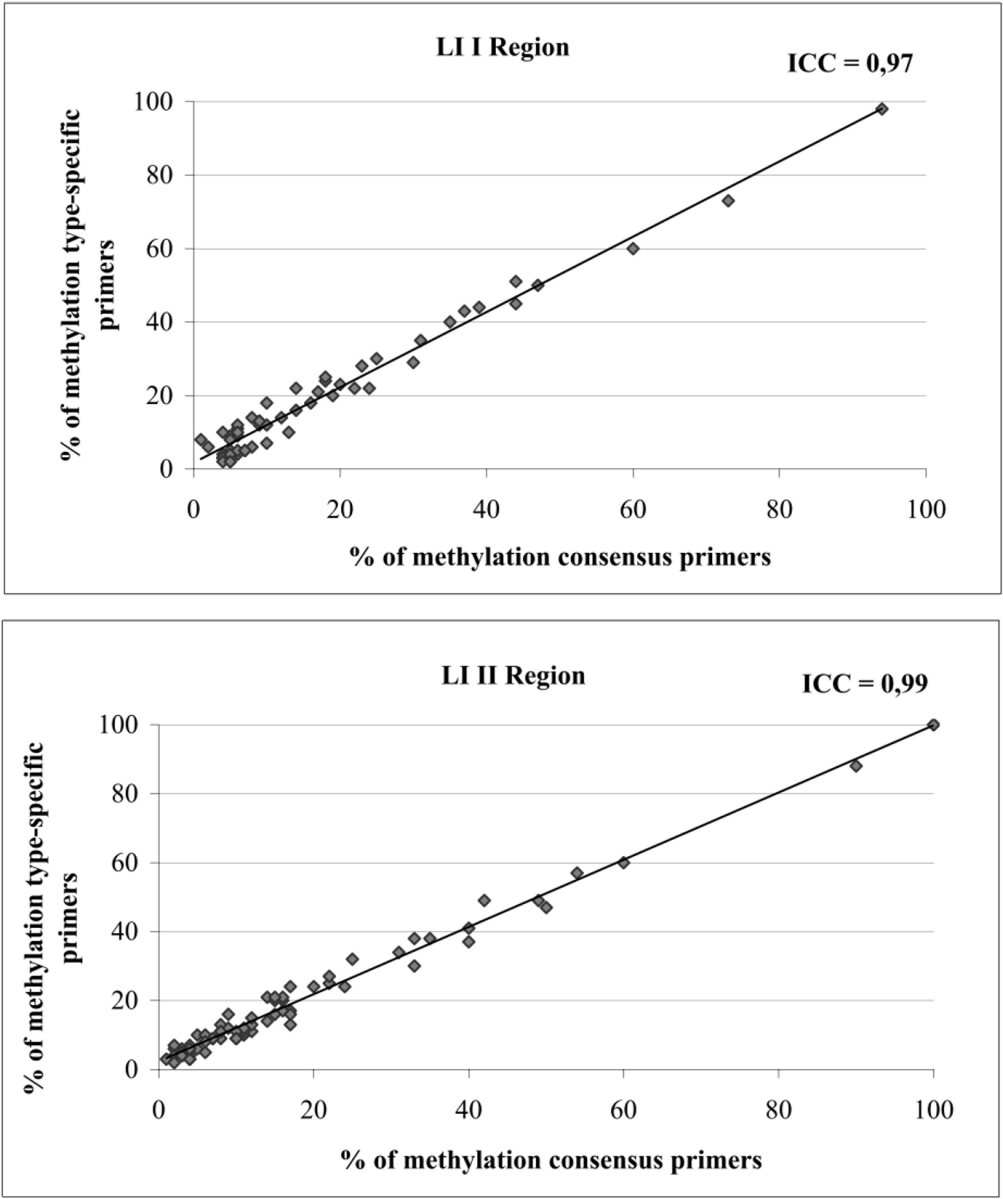
Correlation of the percentage of methylation between methylation protocols using consensus or type specific primers.

A total of 90 samples from the pilot project were tested with two different assays employing either consensus or type-specific primers in the preliminary PCR preceding pyrosequencing. The comparison, carried out for L1 I and L1 II regions and limited to types HPV16, 18, 31, 33, 45, 52, 56 and 58, showed a highly comparable quantification of the mean percentage of methylated CpGs in each region by using the two kinds of primers, with intra-class correlation coefficient between log (methylation) values of 0.97 and 0.99 for L1 I and L1 II respectively (Fig 1 and S1 Table).

### Methylation protocol reproducibility

**Fig 2:**
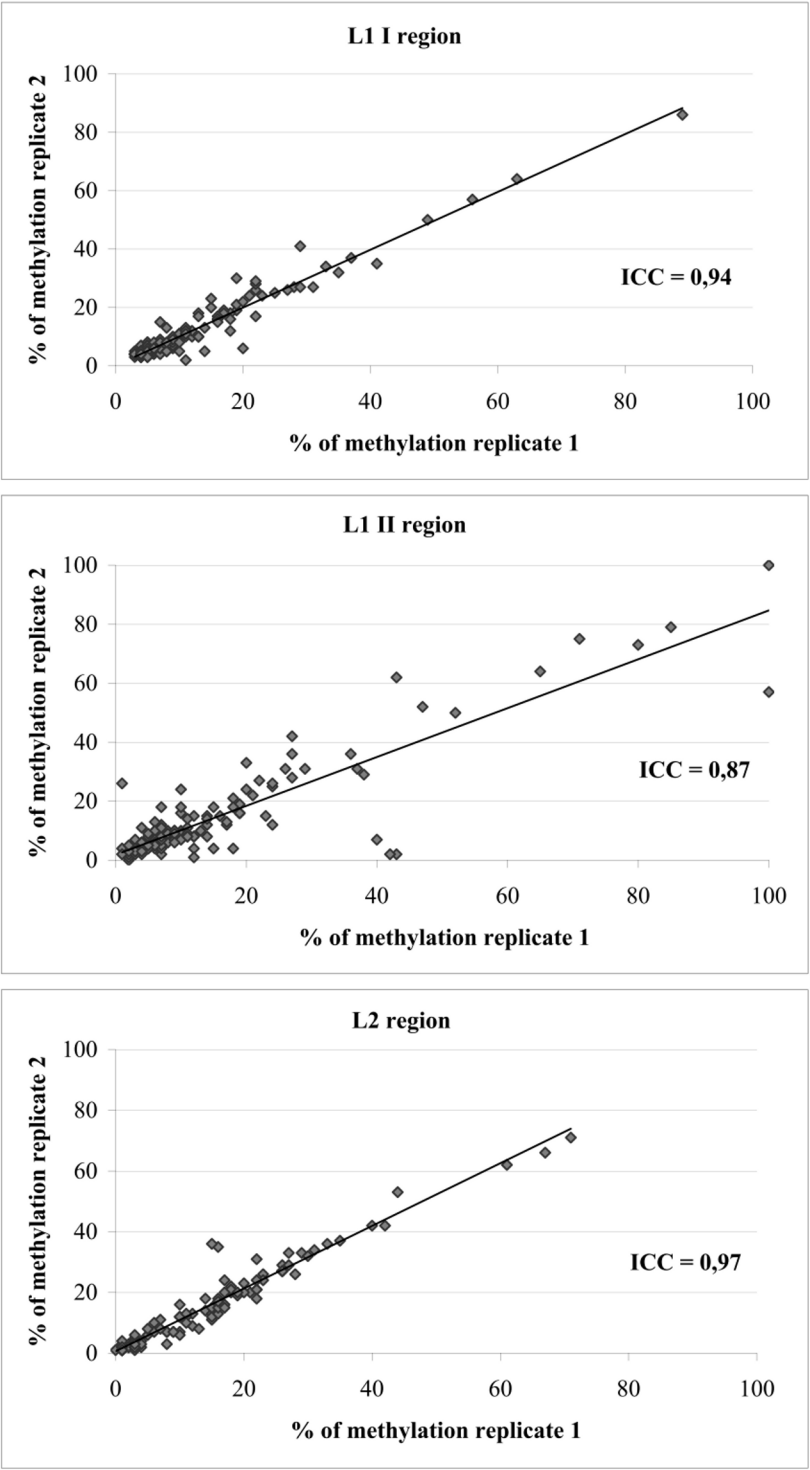
Correlation of the percentage of methylation between the two replicates for each evaluated hrHPV region. The same bisulfite modified DNA samples (N = 140) were used to test methylation on HPV L1 I, L1 II and L2 regions. For L2 regions only 105 had sufficient material for replicates.

**Fig 3:**
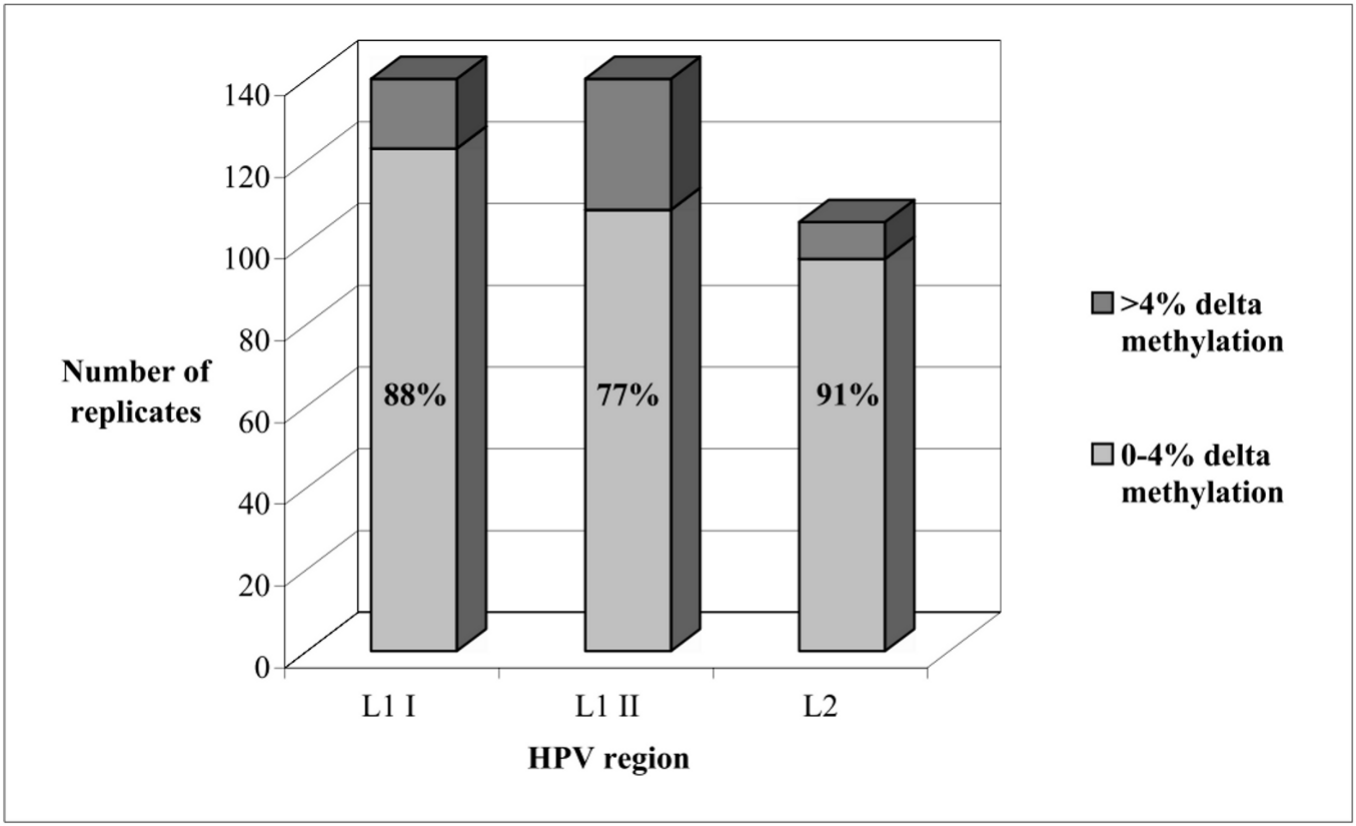
Delta methylation percentage between replicates. The same bisulfite modified DNA samples (N = 140) were used to test methylation on HPV L1 I, L1 II and L2 regions. For L2 regions only 105 had sufficient material for replicates.

Fig 2 shows the plot of replicate results on the original scale. The intra-class correlation coefficient between log (methylation) values was 0.94 for the L1 I, 0.87 for the L1 II and 0.97 for the L2 region. DNA samples gave a 0–4% difference between the two quantitative methylation results in 123/140 (88%) couples of tests for L1 I, in 108/140 (77%) for L1 II and in 96/105 (91%) for L2 (Fig 3). Therefore, one determination was deemed sufficient to obtain a reliable result by methylation quantitative analysis when testing the L1 I and L2 regions, whereas two replicates, taking their average value as final result, were judged to be advisable for the L1 II region.

### Methylation levels and cytology

Cytology was available for the 126 samples listed in S3 Table. The mean percentage of methylation was 23,7% for L1 I, 23,7% for L1 II and 22,7% for L2 in HSIL, versus 16,1% for L1 I, 16,1% for L1 II and 11,5% for L2 in non-HSIL. The difference was statistically significant for L2 (p = 0.0064 by Wilcoxon test) but not for L1 I (p = 0.17) and L1 II (p = 0.24).

## Discussion

We sought the feasibility to design consensus primers for hrHPV methylation analysis of all hrHPV types. Although we targeted the highly conserved HPV capsidic genes, homology among viral types was not sufficient to allow simultaneous testing of all the twelve hrHPV types recognized as oncogenic for cervical mucosas. Nevertheless, we succeeded in the generation of consensus primers able to target two regions on the L1 gene (L1 I and II) for groups of HPV types, aggregated according to their phylogenetic affinity. Generation of consensus primers was instead hampered in the L2 region, due to high density of CpGs and low sequence homology, thus forcing the use of type-specific primers to methylation analyses.

The analytical specificity of the assays newly developed in this study may be in theory ensured by consistency between the expected sequence to analyse and the resulting pyrogram for each hrHPV type. Indeed, when targets different from those expected for the specific genotype were sequenced, failed or confused pyrograms were obtained. To further explore if the use of our consensus primers could provide results systematically different from those obtained with type-specific primers we carried out a comparison between the methylation levels measured starting with type-specific primers and with our consensus primers. The very high agreement obtained supported the analytical reliability of the consensus primers driven assays.

It must be taken into account that samples of lower quality, having undergone repeated freeze and thaw episodes, were used to evaluate assays reproducibility. Despite low quality of these samples, however, good levels of reproducibility of the methylation quantitation by consensus primers were obtained with replicates for the L1 I and L2 regions, with ICC values above 90%. In addition, the difference between replicates was ≤4% in about 90% of cases. With such difference, would the average between replicates be calculated, then the difference between such average and the original results would be ≤2%, an acceptable value in line with other reports [32]. Reproducibility level was lower for the L1 II region. Therefore, two determinations, with a maximum 4% delta in methylation percentage, seem advisable.

The analytical sensitivity of pyrosequencing allows to target viral DNA also in samples with low levels of positivity on the original HC2 screening test. The available DNA amount can marginally affect pyrosequencing results, at most leading to low peaks in the pyrograms, but does not affect the reliability of the methylation count at the single CpG site analysed. In preliminary PCR for pyrosequencing analyses a minimum of 1–10 ng of DNA is conventionally used. However, a standardization of DNA amount is not mandatory [33] as pyrosequencing quantifies the percentage of methylated and unmethylated bases at each position [34]. Indeed, a consensus on a gold standard method for methylation analyses has currently not yet been achieved [20,35].

The methylation protocols were developed and set up on DNA from cervical samples stored for short-time in STM (HPV pilot project sample series) and having undergone only one thaw, for genotyping assay. Nevertheless, the newly developed protocols showed a good performance also with the samples from the Turin NTCC biobank, which had a long-time storage and had been already subjected to multiple freezing and thawing episodes. They performed efficiently with DNA extracted from cervical cells originally collected and stored either in STM or in PreservCyt transport mediums. Even better efficiency is expected in clinical setting by using fresh material.

We considered only samples with single HPV infection, as others before [15]. Taking into account that there is a main interest in testing women with persistent HPV infection, who are at higher risk to develop hg CIN, this choice was done for several reasons. First, single infections, when persistent, have higher methylation levels than multiple-type infections [3]. Second, single infections by oncogenic types were found in 90% of hgCIN [36] and their persistence is reported as more frequently resulting in hgCIN and cancer than with multiple infections [37,38]. Third, methylation protocols using consensus primers could result in overlying sequencing and confused pyrograms when multiple types are present. In different studied cohorts [31,39,40], attribution of HPV types to hgCIN was calculated and top types highlighted. Our methylation protocols with consensus primers targeting L1 I and L1 II regions, respectively, would cover with one PCR for each hrHPV family most of the top types ranked in those studies as prevalent in CIN3.

Our validation of the method was substantially analytical. In a first, very rough, clinical evaluation, we also compared, in the specimens used for developing and setting up the novel assays, the methylation value obtained by our method to cytology, an imprecise predictor of histology. Point estimates suggested an association but, given the small size, the difference was statistically significant only for one region (L2). In-depth analyses to investigate viral methylation association with histologically confirmed current or future hgCIN detection have been conducted in a wide controlled cohort and will be described elsewhere.

## Conclusions

This is, to our knowledge, the first attempt to develop consensus primers for multiple HPV genotypes to use in the assessment of methylation of the L1 and L2 viral regions by pyrosequencing in cervical scrape samples.

We designed and set up such primers and the overall procedure, and assessed their analytical performance in providing a reproducible quantitative estimate similar to that obtained by type-specific PCR. Such data show that the developed consensus primers can be considered equivalent to type-specific primers in order to provide a quantitative evaluation of the methylation of the considered CpGs by pyrosequencing.

HPV methylation protocols covering the majority of oncogenic types could provide an efficient risk stratification tool to quantify viral methylation in HPV positive women.

## Supporting information

S1 Table Comparison of methylation assessment using consensus and type specific primers. (PDF)

S2 Table Single type dedicated primers sequences and annealing temperatures for L1 I and L1 II regions. (PDF)

S3 Table Quantitative methylation in tested samples. (PDF)

S1 Fig Examples of pyrograms with the correspondent sequence to analyse for HPV type and genomic region. (PDF)
