# Supplementary material for: Assessment of viral methylation levels for high risk HPV types by newly designed consensus primers PCR and pyrosequencing
Source: PLoS One. 2018 Mar 26;13(3):e0194619. doi: 10.1371/journal.pone.0194619 (PMC5868804; doi:10.1371/journal.pone.0194619)
Supplement: S1 Table — (PDF) [file pone.0194619.s001.pdf]

S1 Table. Comparison of methylation assessment using consensus and type specific primers

A. HPV region L1 I

ID LAB (N=55)	HPV type	% Methylation Consensus Primers L1 I	% Methylation Type Specific Primers L1 I
1	16	23	28
2	16	9	12
3	16	47	50
4	16	6	11
5	16	5	9
6	16	16	18
7	16	39	44
8	16	5	5
9	16	20	23
10	16	9	13
11	16	10	18
12	16	31	35
14	16	6	12
15	16	37	43
16	16	25	30
17	16	10	12
18	16	12	14
19	16	60	60
20	16	44	51
21	16	14	16
36	31	6	4
37	31	5	2
38	31	4	4
39	31	4	3
40	31	4	3
42	31	5	3
43	31	5	4
44	31	6	5
45	31	8	6
46	31	4	2
48	31	7	5
49	31	5	2
50	31	7	5
51	31	13	10
60	45	73	73
61	45	94	98
62	45	18	24
64	45	35	40
65	45	24	22
67	45	19	20
68	45	14	22
69	45	44	45
70	45	30	29
87	58	6	9
88	58	17	21
89	58	22	22
90	58	6	10
91	58	8	14
92	58	2	6
93	58	5	8
94	58	9	13
95	58	4	10
96	58	1	8
97	58	18	25
98	58	10	7

B. HPV region L1 II

ID LAB (N=68)	HPV type	% Methylation Consensus Primers L1 II	% Methylation Type Specific Primers L1 II
1	16	17	24
2	16	5	10
3	16	42	49
4	16	8	9
5	16	3	6
6	16	8	11
8	16	2	6
9	16	2	7
10	16	8	13
11	16	14	14
12	16	31	34
14	16	7	9
15	16	25	32
16	16	22	25
17	16	6	10
18	16	1	3
20	16	17	17
21	16	4	7
22	18	17	13
23	18	16	20
24	18	12	11
26	18	2	4
27	18	33	38
28	18	5	6
30	18	16	17
31	18	10	9
32	18	20	24
33	18	6	5
34	18	3	4
35	18	11	10
52	33	12	13
53	33	2	2
54	33	7	9
55	33	100	100
56	33	14	21
57	33	100	100
59	33	9	16
60	45	54	57
61	45	90	88
65	45	11	11
69	45	40	37
70	45	33	30
71	52	15	16
72	52	9	12
73	52	11	12
74	52	10	11
76	52	35	38
77	52	15	20
78	52	22	27
79	52	50	47
80	52	3	4
81	56	10	9
82	56	24	24
83	56	4	4

84	56	4	3
86	56	4	3
87	58	3	5
88	58	8	11
89	58	16	21
90	58	3	4
91	58	15	21
92	58	4	6
93	58	6	8
94	58	12	15
95	58	17	16
97	58	60	60
99	58	40	41
100	58	49	49
