# Supplementary material for: Assessment of viral methylation levels for high risk HPV types by newly designed consensus primers PCR and pyrosequencing
Source: PLoS One. 2018 Mar 26;13(3):e0194619. doi: 10.1371/journal.pone.0194619 (PMC5868804; doi:10.1371/journal.pone.0194619)
Supplement: S2 Table — (PDF) [file pone.0194619.s002.pdf]

S2 Table. Single type dedicated primers sequences and annealing temperatures for L1 I and L1 II regions

Target Region	HPV type	PCR primers and assay conditions			Pyrosequencing primers	
		PCR primer *	Frag ment bp	PCR anneal. T	Sequencing* primer	HPV genomic strand
L1 I[#]	16	Forward GATATTTGAAAAAAAATATGGTA Reverse Bio-AATAACTTTTATTTACATCCTAATTAT	78	45	GATATTTGAAAA AAAATATGGTA	Lower
	31	Forward ATATTTGTAAAAAAATATGAT Reverse bio-AATAACTTTTATTTACATCCTAATTAT	77	45	ATATTTGTAAAA AAATATGAT	Lower
	45	Forward TTTATATTGATATGAGGATATTTTAGG Reverse bio-CCCTATTTTTTTACAAATAACTTTATAAC	232	50	TTATAGAAGGTG GTGGAAGAT	Lower
	58	Forward GGTATAGGAGGTAGGTATATAGTGGT Reverse bio-AATCCATTTATTCCTATATCTCCACT	180	60	GATATTTGTAAA AAAATATGGAA	Lower
L1 II[§]	16	Forward GTTTGTTAGATATTTATTTAATAGGGTTGG Reverse bio-ATTACCCCAACAAATACCATTA	217	55	TGTTGGTGAAAA TGTATTAG	Upper
	18	Forward GTTTGTAGATTTTTATGGGGATTTTATG Reverse bio-ACCAACAAATACCATTATTATAACCCT	266	50	ATATATTAAAGG TATAGGTATG	Upper
	33	Forward GGTATATTAGGAGAGGTTGT Reverse bio-ATTACCCCAACAAATACCATTA	189	60	GGTATATTAGGA GAGGTTGT	Upper
	45	Forward AGATTTATATATTAAAGGTATTAG Reverse bio-ACCAACAAATACCATTATTATAACCCT	164	54	AGATTTATATAT TAAAGGTATTAG	Upper
	52	Forward GTTAGATATTTTTTTAATAGGG Reverse bio-ATTACCCCAACAAATACCATTA	219	57	GTTAGATATTTT TTTAATAGGG	Upper
	56	Forward GAGAATTTTTTTTTAGTTTTGTATA Reverse bio-ATTACCCCAACAAATACCATTA	134	57	GAGAATTTTTTT TTAGTTTTGTAT A	Upper
	58	Forward TAGGGTTGGAAAATTTGG Reverse bio-ATTACCCCAACAAATACCATTA	196	60	TAGGGTTGGAAA ATTTGG	Upper

* Bisulfite modified sequences
[#] L1 I. HPV L1 gene. Reference HPV16 CpG: 5601, 5606, 5609, 5616
[§] L1 II. HPV L1 gene. Reference HPV16 CpG: 6457
