# Supplementary material for: Assessment of viral methylation levels for high risk HPV types by newly designed consensus primers PCR and pyrosequencing
Source: PLoS One. 2018 Mar 26;13(3):e0194619. doi: 10.1371/journal.pone.0194619 (PMC5868804; doi:10.1371/journal.pone.0194619)
Supplement: S3 Table — (PDF) [file pone.0194619.s003.pdf]

## S3 Table. Quantitative methylation in tested samples

| Sample[a] | HPV family | HPV type | Cytology[b] | Mean % methyl L1 I[c] | PyroMark Q24 score[d] | Mean % methyl L1 II[e] | PyroMark Q24 score[d] | Mean % methyl L2[f] | PyroMark Q24 score[d] |
|---|---|---|---|---|---|---|---|---|---|
| 1 | 16 | 16 | NILM | 5 | passed | 10 | Passed | 16 | Check |
| 2 | 16 | 16 | HSIL | 8 | passed | 3 | Passed | 5 | Passed |
| 3 | 16 | 16 | HSIL | 5 | Passed | 7 | Passed | 4 | Passed |
| 4 | 16 | 16 | HSIL | 47 | Passed | 48 | Passed | 38 | Passed |
| 5 | 16 | 16 | HSIL | 10 | Passed | 12 | Passed | 11 | Passed |
| 6 | 16 | 16 | HSIL | 12 | Passed | 18 | Passed | 11 | Passed |
| 7 | 16 | 16 | HSIL | 9 | Passed | 6 | Passed | 6 | Passed |
| 8 | 16 | 16 | HSIL | 19 | Passed | 12 | Passed | 35 | Check |
| 9 | 16 | 16 | HSIL | 20 | Passed | 21 | Passed | 6 | Passed |
| 10 | 16 | 16 | NILM | 22 | Passed | 4 | Passed | 10 | Check |
| 11 | 18 | 18 | NILM | 26 | Passed | 9 | Passed | 12 | Passed |
| 12 | 18 | 18 | LSIL | 34 | Passed | 31 | Check | 26 | Passed |
| 13 | 18 | 18 | NILM | 25 | Passed | 26 | Check | 22 | Passed |
| 14 | 18 | 18 | LSIL | 20 | Passed | 12 | Passed | 21 | Passed |
| 15 | 18 | 18 | NILM | 14 | Passed | 5 | Check | 4 | Check |
| 16 | 18 | 18 | NILM | 18 | Passed | 16 | Passed | 9 | Check |
| 17 | 18 | 18 | LSIL | 11 | Passed | 15 | Passed | 5 | Passed |
| 18 | 18 | 18 | ASCUS | 29 | Passed | 20 | Passed | 22 | Check |
| 19 | 18 | 18 | NILM | 20 | Passed | 13 | Passed | 9 | Passed |
| 20 | 18 | 18 | NILM | 26 | Passed | 34 | Check | 5 | Passed |
| 21 | 16 | 31 | NILM | 13 | Passed | 8 | Passed | 14 | Check |
| 22 | 16 | 31 | NILM | 5 | Passed | 4 | Passed | 4 | Passed |
| 23 | 16 | 31 | HSIL | 6 | Passed | 5 | Passed | 4 | Passed |
| 24 | 16 | 31 | HSIL | 10 | Passed | 7 | Passed | 14 | Passed |
| 25 | 16 | 31 | HSIL | 30 | Check | 29 | Passed | 24 | Check |
| 26 | 16 | 31 | HSIL | 18 | Check | 7 | Passed | 11 | Passed |
| 27 | 16 | 31 | HSIL | 19 | Check | 9 | Passed | 23 | Check |
| 28 | 16 | 31 | HSIL | 8 | Passed | 6 | Passed | 7 | Passed |
| 29 | 16 | 31 | HSIL | 14 | Passed | 12 | Passed | 16 | Check |
| 30 | 16 | 31 | LSIL | 9 | Passed | 12 | Passed | 10 | Check |
| 31 | 16 | 33 | NILM | 19 | Passed | 10 | Passed | 29 | Passed |
| 32 | 16 | 33 | LSIL | 7 | Passed | 19 | Passed | 11 | Passed |
| 33 | 16 | 33 | NILM | 10 | Passed | 13 | Passed | 13 | Passed |
| 34 | 16 | 33 | NILM | 18 | Check | 2 | Passed | 3 | Passed |
| 35 | 16 | 33 | NILM | 6 | Passed | 7 | Passed | 12 | Passed |
| 36 | 16 | 33 | NILM | 4 | Passed | 2 | Passed | 3 | Passed |
| 37 | 16 | 33 | HSIL | 12 | Passed | 61 | Passed | 26 | Passed |
| 38 | 16 | 33 | ASCUS | 5 | Passed | 2 | Passed | 9 | Passed |
| 39 | 16 | 33 | HSIL | 34 | Passed | 30 | Passed | 56 | Passed |
| 40 | 16 | 33 | NILM | 16 | Check | 40 | Passed | 52 | Passed |
| 41 | 16 | 35 | LSIL | 3 | Check | 3 | Passed | 1 | Passed |
| 42 | 16 | 35 | NILM | 11 | Passed | 4 | Passed | 3 | Passed |
| 43 | 16 | 35 | NILM | 8 | Passed | 11 | Passed | 7 | Passed |
| 44 | 16 | 35 | NILM | 9 | Passed | 9 | Check | 1 | Passed |
| 45 | 16 | 35 | NILM | 6 | Passed | 13 | Passed | 5 | Passed |

| Sample[a] | HPV family | HPV type | Cyto logy[b] | Mean % methyl L1 I[c] | PyroMark Q24 score[d] | Mean % methyl L1 II[e] | PyroMark Q24 score[d] | Mean % methyl L2[f] | PyroMark Q24 score[d] |
|---|---|---|---|---|---|---|---|---|---|
| 46 | 16 | 35 | NILM | 6 | Check | 6 | Passed | 1 | Passed |
| 47 | 16 | 35 | NILM | 5 | Passed | 6 | Passed | 1 | Passed |
| 48 | 16 | 35 | HSIL | 27 | Passed | 12 | Passed | 26 | Passed |
| 49 | 16 | 35 | LSIL | 3 | Passed | 1 | Passed | 1 | Passed |
| 50 | 16 | 35 | LSIL | 7 | Check | 2 | Passed | 2 | Passed |
| 51 | 18 | 39 | NILM | 43 | Check | 10 | Passed | 14 | Passed |
| 52 | 18 | 39 | LSIL | 11 | Passed | 10 | Passed | 6 | Passed |
| 53 | 18 | 39 | NILM | 24 | Passed | 9 | Passed | 10 | Passed |
| 54 | 18 | 39 | NILM | 12 | Passed | 7 | Passed | 3 | Passed |
| 55 | 18 | 39 | NILM | 10 | Passed | 10 | Passed | 8 | Passed |
| 56 | 18 | 39 | NILM | 30 | Check | 18 | Passed | 1 | Passed |
| 57 | 18 | 39 | LSIL | 15 | Passed | 8 | Passed | 18 | Passed |
| 58 | 18 | 39 | LSIL | 8 | Passed | 7 | Passed | 6 | Passed |
| 59 | 18 | 39 | NILM | 7 | Passed | 8 | Passed | 6 | Passed |
| 60 | 18 | 39 | NILM | 12 | Passed | 11 | Check | 16 | Passed |
| 61 | 18 | 45 | NILM | 73 | Passed | 55 | Passed | 18 | Passed |
| 62 | 18 | 45 | NILM | 43 | Passed | 41 | Passed | 8 | Passed |
| 63 | 18 | 45 | NILM | 26 | Passed | 28 | Passed | 9 | Passed |
| 64 | 18 | 45 | NILM | 52 | Passed | 46 | Passed | 21 | Check |
| 65 | 18 | 45 | HSIL | 92 | Passed | 80 | Passed | 86 | Passed |
| 66 | 18 | 45 | HSIL | 94 | Passed | 89 | Passed | 88 | Passed |
| 67 | 18 | 45 | NILM | 24 | Passed | 6 | Passed | 5 | Passed |
| 68 | 18 | 45 | NILM | 28 | Passed | 22 | Passed | 16 | Passed |
| 69 | 18 | 45 | NILM | 24 | Passed | 25 | Check | 15 | Passed |
| 70 | 18 | 45 | HSIL | 18 | Passed | 11 | Passed | 5 | Passed |
| 71 | 18 | 45 | NILM | 30 | Passed | 28 | Passed | 13 | Passed |
| 72 | 18 | 51 | NILM | 11 | Check | 23 | Passed | 6 | Passed |
| 73 | 18 | 51 | LSIL | 3 | Passed | 7 | Passed | 2 | Passed |
| 74 | 18 | 51 | NILM | 8 | Passed | 28 | Passed | 19 | Passed |
| 75 | 18 | 51 | LSIL | 5 | Check | 4 | Passed | 2 | Passed |
| 76 | 18 | 51 | NILM | 17 | Passed | 28 | Passed | 10 | Passed |
| 77 | 18 | 51 | LSIL | 5 | Check | 8 | Passed | 4 | Passed |
| 78 | 18 | 51 | NILM | 25 | Passed | 78 | Passed | 17 | Check |
| 79 | 18 | 51 | NILM | 4 | Passed | 18 | Passed | 4 | Passed |
| 80 | 18 | 51 | NILM | 5 | Check | 20 | Passed | 1 | Passed |
| 81 | 18 | 51 | NILM | 21 | Check | 19 | Passed | 4 | Passed |
| 82 | 18 | 51 | NILM | 1 | Passed | 11 | Check | 2 | Passed |
| 83 | 16 | 52 | NILM | 14 | Passed | 2 | Check | 16 | Passed |
| 84 | 16 | 52 | NILM | 18 | Passed | 44 | Passed | 6 | Passed |
| 85 | 16 | 52 | NILM | 7 | Passed | 8 | Passed | 1 | Passed |
| 86 | 16 | 52 | NILM | 10 | Passed | 14 | Passed | 5 | Passed |
| 87 | 16 | 52 | NILM | 24 | Passed | 27 | Passed | 6 | Passed |
| 88 | 16 | 52 | NILM | 24 | Passed | 38 | Passed | 17 | Passed |
| 89 | 16 | 52 | HSIL | 10 | Passed | 21 | Passed | 9 | Passed |
| 90 | 16 | 52 | NILM | 24 | Passed | 43 | Passed | 7 | Passed |
| 91 | 16 | 52 | NILM | 18 | Passed | 41 | Passed | 4 | Check |
| 92 | 16 | 52 | HSIL | 24 | Passed | 39 | Passed | 11 | Passed |

| Sample[a] | HPV family | HPV type | Cytology[b] | Mean % methyl L1 I[c] | PyroMark Q24 score[d] | Mean % methyl L1 II[e] | PyroMark Q24 score[d] | Mean % methyl L2[f] | PyroMark Q24 score[d] |
|---|---|---|---|---|---|---|---|---|---|
| 93 | 16 | 56 | NILM | 14 | Passed | 7 | Passed | 39 | Passed |
| 94 | 16 | 56 | NILM | 23 | Passed | 11 | Check | 1 | Passed |
| 95 | 16 | 56 | ASCUS | 26 | Passed | 14 | Passed | 42 | Passed |
| 96 | 16 | 56 | LSIL | 4 | Passed | 4 | Passed | 2 | Passed |
| 97 | 16 | 56 | NILM | 13 | Passed | 7 | Passed | 8 | Passed |
| 98 | 16 | 56 | NILM | 11 | Passed | 7 | Passed | 15 | Passed |
| 99 | 16 | 56 | LSIL | 5 | Passed | 12 | Passed | 3 | Passed |
| 100 | 16 | 56 | NILM | 5 | Passed | 48 | Check | 5 | Passed |
| 101 | 16 | 56 | NILM | 4 | Passed | 7 | Passed | 3 | Passed |
| 102 | 16 | 56 | NILM | 18 | Passed | 9 | Passed | 18 | Check |
| 103 | 16 | 56 | NILM | 17 | Passed | 19 | Passed | 30 | Passed |
| 104 | 16 | 56 | NILM | 5 | Passed | 4 | Passed | 4 | Check |
| 105 | 16 | 56 | NILM | 13 | Passed | 11 | Passed | 1 | Check |
| 106 | 16 | 56 | NILM | 31 | Check | 41 | Passed | 43 | Passed |
| 107 | 16 | 58 | NILM | 19 | Passed | 13 | Passed | 32 | Passed |
| 108 | 16 | 58 | LSIL | 6 | Passed | 3 | Passed | 12 | Passed |
| 109 | 16 | 58 | NILM | 12 | Passed | 8 | Passed | 21 | Passed |
| 110 | 16 | 58 | NILM | 67 | Passed | 65 | Check | 75 | Passed |
| 111 | 16 | 58 | NILM | 10 | Passed | 6 | Passed | 16 | Passed |
| 112 | 16 | 58 | NILM | 7 | Check | 5 | Passed | 21 | Passed |
| 113 | 16 | 58 | NILM | 16 | Passed | 14 | Passed | 24 | Passed |
| 114 | 16 | 58 | NILM | 16 | Passed | 9 | Passed | 35 | Passed |
| 115 | 16 | 58 | NILM | 10 | Passed | 5 | Passed | 27 | Passed |
| 116 | 16 | 58 | NILM | 10 | Passed | 7 | Passed | 12 | Passed |
| 117 | 18 | 59 | NILM | 18 | Passed | 11 | Passed | 2 | Passed |
| 118 | 18 | 59 | NILM | 15 | Passed | 14 | Passed | 2 | Passed |
| 119 | 18 | 59 | LSIL | 15 | Passed | 19 | Passed | 2 | Passed |
| 120 | 18 | 59 | LSIL | 11 | Passed | 11 | Passed | 2 | Passed |
| 121 | 18 | 59 | NILM | 17 | Passed | 8 | Passed | 4 | Passed |
| 122 | 18 | 59 | NILM | 37 | Passed | 31 | Passed | 4 | Passed |
| 123 | 18 | 59 | NILM | 11 | Passed | 10 | Passed | 3 | Passed |
| 124 | 18 | 59 | LSIL | 11 | Passed | 14 | Passed | 2 | Passed |
| 125 | 18 | 59 | NILM | 17 | Passed | 10 | Passed | 2 | Passed |
| 126 | 18 | 59 | NILM | 12 | Passed | 11 | Passed | 1 | Passed |
| HPV16 Pl. U [g] | 16 | 16 | - | 4 (1-6) | Passed | 3 (3-7) | Passed | 2 (2-3) | Passed |
| HPV18 Pl. U [g] | 18 | 18 | - | 5 (4-9) | Passed | 5 (3-9) | Passed | 3 (1-6) | Passed |
| HPV16 Pl. M [g] | 16 | 16 | - | 93 (88-95) | Passed | 95 (93-96) | Passed | 87 (85-92) | Passed |
| HPV18 Pl. M [g] | 18 | 18 | - | 84 (80-86) | Passed | 86 (80-96) | Passed | 94 (90-95) | Passed |
| SiHA [g] | 16 | 16 | - | 92 (90-93) | Passed | 98 (97-98) | Passed | na | - |
| HeLA [g] | 18 | 18 | - | nd | - | 48 (45-57) | Passed | nd | - |

[a]Samples collected in STM from women who participated at the pilot project in Turin.

[b]NILM, Negative for Intraepithelial Lesion or Malignancy; ASCUS Atypical Squamous Cells of Undetermined Significance; LSIL, Low-Grade Squamous Intraepithelial Lesion; HSIL, High-Grade Squamous Intraepithelial Lesion.

[c] Mean percentage of methylation among all the target CpGs in the L1 I region released by PyroMark Q24 software (reference HPV16 CpG 5601, 5606, 5609, 5016)

[d] Colour-based score for result quality: Passed,(blue); Check, (yellow); Failed (red). No red score was assigned by the automatic PyroMark Q24 software.

[e] Percentage of methylation released by PyroMark Q24 software for the targeted CpG in the L1 II region (reference HPV16 CpG 6457). For HPV56 and HPV 58 mean percentage of methylation between the two targeted CpGs is reported

[f] Percentage of methylation released by PyroMark Q24 software for the targeted CpG in the L2 region (reference HPV16 CpG 4261)

[g] Mean (and range) of at least ten replicates: Pl=plasmid; U= unmethylated; M=methylated; na=not applicable, for L2 only synthetic plasmids were used as controls; nd = not detectable for deletion upon virus integration [*Schwartz E, et al. Structure and transcription of human papillomavirus sequences in cervical carcinoma cells. Nature 1995;314: 111-114*]
