# Supplementary material for: Assessment of viral methylation levels for high risk HPV types by newly designed consensus primers PCR and pyrosequencing
Source: PLoS One. 2018 Mar 26;13(3):e0194619. doi: 10.1371/journal.pone.0194619 (PMC5868804; doi:10.1371/journal.pone.0194619)
Supplement: S1 Fig — (PDF) [file pone.0194619.s004.pdf]

S1 Fig. Examples of pyrograms with the correspondent sequence to analyse for HPV type and genomic region.

A. L1 I region, type HPV16

AAYGTTTAYGTYGTTTTYGTAATATGTAATAATTAGGATGTAA

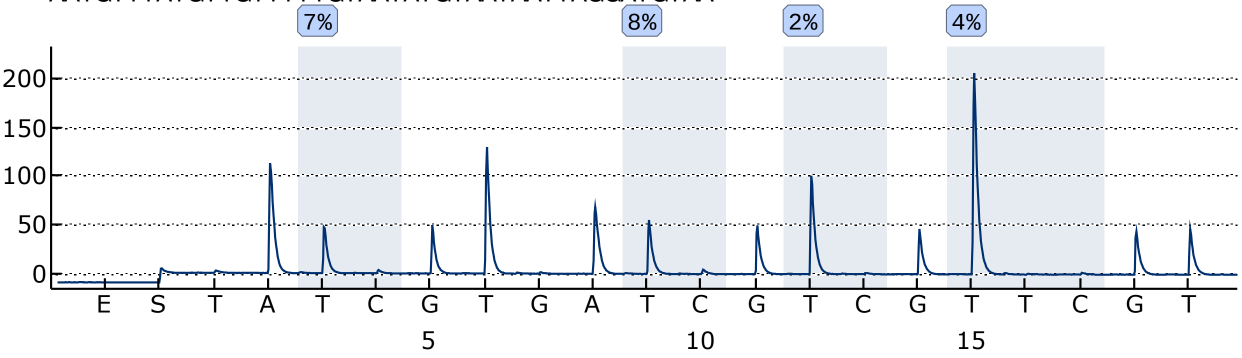

B. L1 II region, type HPV16

AYGATTTATATATTAAAGGTTTTGGGT

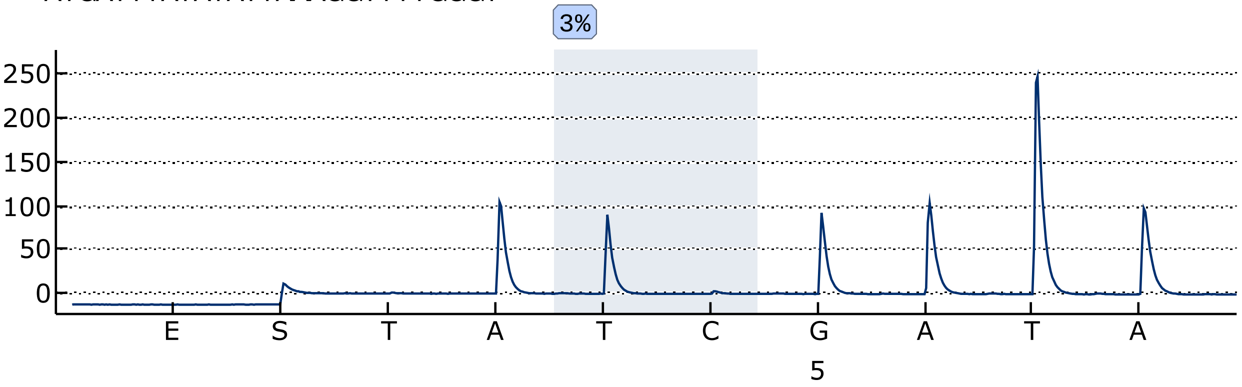

C. L2 region, type HPV16

TA/GGTYGATGTAYGTTTTGTGYGTTT

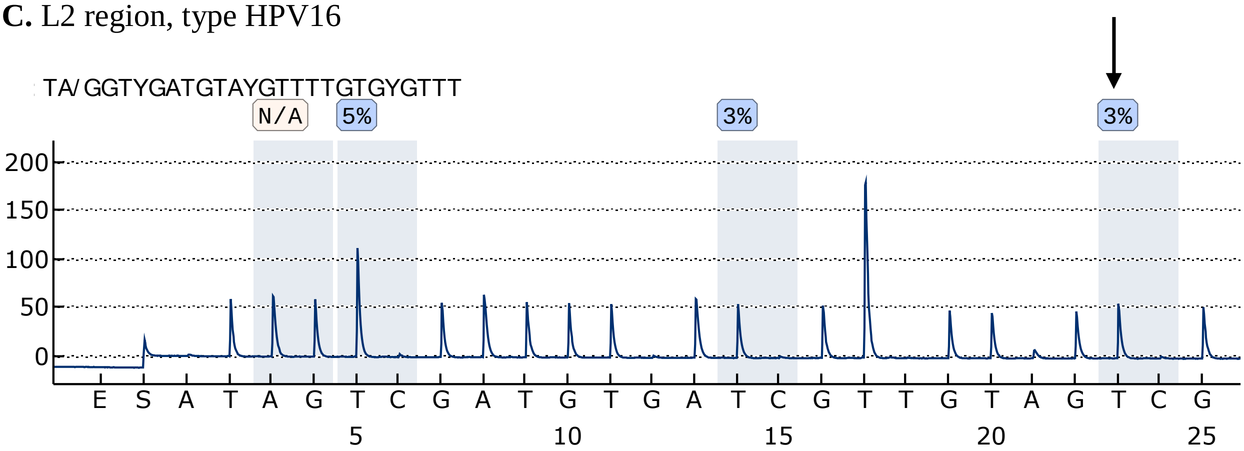

D. L1 I region, type HPV18

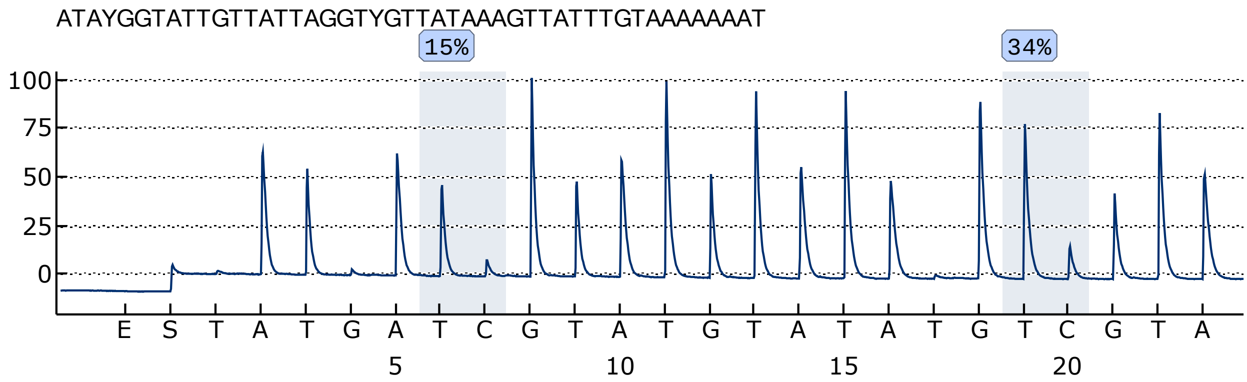

E. L1 II region, type HPV18

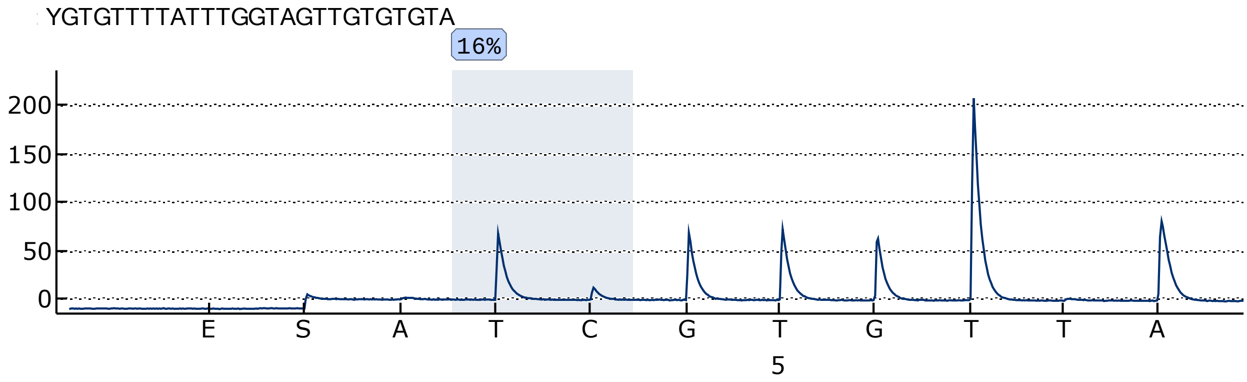

F. L2 region, type HPV18

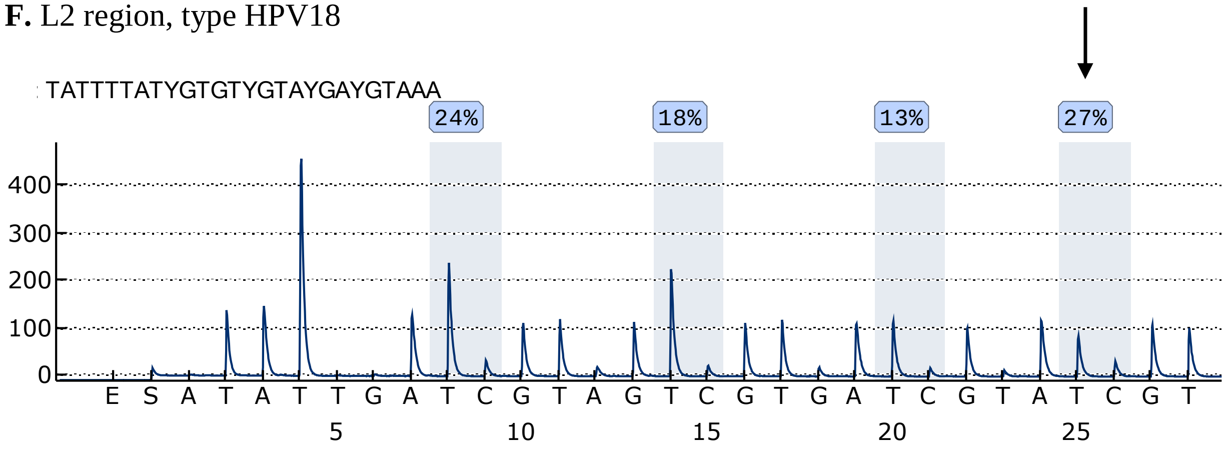

C, F: Arrows indicate the target CpG, as several CpGs are present in the sequence to analyse.
